# A brief report on the relationship between self-control, video game addiction and academic achievement in normal and ADHD students

**DOI:** 10.1556/JBA.2.2013.4.7

**Published:** 2013-12-13

**Authors:** Maryam Haghbin, Fatemeh Shaterian, Davood Hosseinzadeh, Mark D. Griffiths

**Affiliations:** ^1^Department of Educational Psychology, Saveh Branch, Islamic Azad University, Saveh, Iran; ^2^Nottingham Trent University, United Kingdom

**Keywords:** video game addiction, self-control, academic achievement, gender, ADHD students

## Abstract

*Background and aims:* Over the last two decades, research into video game addiction has grown increasingly. The present research aimed to examine the relationship between video game addiction, self-control, and academic achievement of normal and ADHD high school students. Based on previous research it was hypothesized that (i) there would be a relationship between video game addiction, self-control and academic achievement (ii) video game addiction, self-control and academic achievement would differ between male and female students, and (iii) the relationship between video game addiction, self-control and academic achievement would differ between normal students and ADHD students. *Methods:* The research population comprised first grade high school students of Khomeini-Shahr (a city in the central part of Iran). From this population, a sample group of 339 students participated in the study. The survey included the Game Addiction Scale (Lemmens, Valkenburg & Peter, 2009), the Self-Control Scale (Tangney, Baumeister & Boone, 2004) and the ADHD Diagnostic checklist (Kessler et al., 2007). In addition to questions relating to basic demographic information, students’ Grade Point Average (GPA) for two terms was used for measuring their academic achievement. These hypotheses were examined using a regression analysis. *Results:* Among Iranian students, the relationship between video game addiction, self-control, and academic achievement differed between male and female students. However, the relationship between video game addiction, self-control, academic achievement, and type of student was not statistically significant. *Conclusions:* Although the results cannot demonstrate a causal relationship between video game use, video game addiction, and academic achievement, they suggest that high involvement in playing video games leaves less time for engaging in academic work.

## INTRODUCTION

In all educational systems around the world, the level of students' academic achievement serves as one of the success indicators of their educational activities. Many different factors are involved in academic achievement such as personality and contextual factors. Self-control is considered one of these personality traits. Logue (1995) defines self-control as “doing the activities followed by a later but a larger reward.” Self-control can be seen from different points of view. For example, it has been described as a ‘satisfaction effect’ and functionally as the time duration somebody waits to achieve a more valuable but more distant outcome (Rodriguez, 1989; cited by Storey, 2002). People use self-control when they have decided to achieve a long-term goal. For such an achievement, a person may dismiss the pleasure of eating, drinking alcohol, gambling, spending money, staying awake and/or sleeping. In many difficult and conflicting situations where making a choice is required, people are recommended to use self-control (Rodriguez, 1989; cited by Storey, 2002). Mobilized with high self-control, students may experience more success in the long route through education.

Compared to those who have low self-control, those with high self-control are more successful during performance of their tasks. Also, they are more able to separate their leisure time activities from the other kinds, take better advantage of their study time, choose more suitable courses and classes, and control activities and entertainments which may be harmful to their educational development. Previous studies evidently indicate that the self-control may enhance the academic achievement. Feldman, Martinez-Pons and Shaham (1995) observed that the children with a higher self-control achieved higher grades in a computer training course. Little research has been carried out on the role of the students' self-control level as a mediating factor in the relationship between personality traits and academic performance (Normandeau & Guay, 1998). Results from Tangney et al. (2004) support the hypothesis that high self-control predicts enhanced academic performance. Furthermore, Duckworth and Seligman (2005) showed that the effect of self-control on the academic achievement is twice that of intelligence.

Flynn (1985) observed a relationship between the academic achievement of the emigrant African-American male students and development due to the delay of gratification. In two concurrent investigations, Mischel, Shoda and Peake (1988), and Shoda, Mischel and Peake (1990), evaluated the capacity of delaying the gratification and mind satisfaction in four-year-old children. They examined the children again after graduating from high school and one further time after graduating from college. They found that the children who were more successful in delaying gratification and mind satisfaction during childhood obtained higher scores as adults. According to Wolfe and Johnson (1995), self-control was the only trait among 32 personality variables that contributed significantly to prediction of university students' GPA (grade point average). Taken as a whole, empirical research shows that the high self-control leads to a better academic achievement (Tangney et al., 2004).

Another activity that can be included as a contextual factor relevant to students' academic achievement is video game addiction. According to a variety of studies, playing video games can affect the academic achievement of children and adolescents (Harris, 2001). Nowadays, playing video games has turned into one of the most time-consuming leisure time activities of children and adolescents and is increasingly taking the place of traditional and interactive games and activities (Frölich, Lehmkuhl & Döpfner, 2009). Despite the many advantages of such technology, computers and computer gaming may negatively affect people’s social skills (Griffiths, 2010a). Video game addiction may lower teenagers’ motivation for communicating with other people and consequently impose negative effects on their social relationships (Kuss & Griffiths, 2012). Furthermore, Huge and Gentile (2003), among others, note that video game addiction may cause a failure in teenagers’ academic performance.

While playing such games, players may forget about everything and get immersed in the game. Video game playing also has the capacity to stop players engaging in other activities (including educational study). Additionally, excessive video gamers are less interested in school. Since too much playing reduces the required time for doing homework, it can consequently negatively affect the individual’s academic achievement (Roe & Muijs, 1998). Some research has shown that the students with low academic achievement spend more time (more than 3 hours a day) playing video games in comparison to those who are academically successful (Benton, 1995). Excessive video gaming may reduce the student’s readiness for attempting at learning and study (Walsh, 2002). However, there is also a lot of empirical evidence showing how video games can enhance students' academic achievement (Griffiths, 2010b).

Chan and Rabinowitz (2006) believe that there is a relationship between ADHD and frequent video gaming. In fact, attention-deficit/hyperactivity disorder is the most prevalent psychiatric disorder among children and adolescents of school age. The behaviour usually leads to a conflict between the student and the school staff as well as the family members. Feelings of desperation and worthlessness can also emerge. Because of the changeability of these children’s behaviour, parents often believe that their children’s annoying behaviors are intentional (Biederman & Faraone, 2004). Due to the symptoms of hyperactivity and attention deficiency, ADHD children are vulnerable to various negative consequences including academic problems, behavioural disorders, and various comorbidity risks. Hence, immediate interventions are required in order to minimize such problems. ADHD is not just a childhood disorder and should not be considered as a periodic disorder. It is chronic and enduring just like many other developmental disorders (Biederman & Faraone, 2004). Such patients will face many consequences at including cognitive-behavioral problems, emotional problems, academic failure, occupational problems and a higher possibility of emerging high-risk drug abuse behaviors (Hervey, Epstein & Curry, 2004).

Given that the video game addiction and the associated issues have been the subject matter of increasing research in clinical, counseling and educational domains, the present research study aimed to examine the relationship between video game addiction, self-control, and academic achievement of both normal and ADHD high school students. Based on previous research it was hypothesized that (i) there would be a relationship between video game addiction, self-control and academic achievement, (ii) the relationship between video game addiction, self-control and academic achievement would differ between male and female students, and (iii) the relationship between video game addiction, self-control and academic achievement would differ between normal students and ADHD students.

## METHODS

### Participants

The research population comprised first grade high school students of Khomeini-Shahr (a city in the central part of Iran). Of this population, a representative group of 339 students participated in the study. Two-stage cluster sampling was used. When data are obtained by two-stage cluster sampling, serious problems may arise if conventional methods that ignore the intracluster correlations are used. Because of this, intracluster correlation was estimated. Eighteen schools were randomly selected from 234 schools in this city. Following this, one class from each school was randomly selected. Thirteen students' questionnaires were omitted from analysis because they were not properly completed leaving a final sample of 326 high school students. Among the students, 146 (49.1%) were females, and 166 (50.9%) were males.

### Materials

– The data were collected via a questionnaire. In addition to questions relating to basic demographic information, students' Grade Point Average (GPA) for two terms was used as a measure of their academic achievement. The questionnaire also included the Game Addiction Scale (Lemmens et al., 2009), the Self-Control Scale (Tangney et al., 2004) and the ADHD Diagnostic checklist (Kessler et al., 2007). In this study, all scales were translated into Persian and back-translated into English by two independent official translators. Comparison of the original version and the back-translated into English version showed that there were only minor changes between the two forms of each scale. Cronbach’s alpha was then used to assess the internal consistency of the instruments using SPSS software. These coefficients are reported below.

– *Computer and Video Game Addiction Scale* (Lemmens et al., 2009): This questionnaire measures seven underlying addiction criteria including salience, tolerance, moodmodification, relapse, withdrawal, conflict and problems. The resulting Cronbach’s alpha coefficients in this sample respectively 0.93, 0.93, 0.69, 0.98, 0.91, 0.88 and 0.99, respectively.

– *Self-Control Scale* (Tangney et al., 2004): This questionnaire measures five factors (self-discipline, resistance to impulsivity, healthy habits, work ethic and reliability). Both the reliability and validity of this questionnaire have been reported to be 0.89.

– *Diagnostic Checklist and Self-report Scale* (Kessler et al., 2007): This scale assesses six hyperactivity criteria as listed in the *Diagnostic and Statistical Manual of Mental Disorders* (American Psychiatric Association, 2000): (i) often fails to give close attention to details or makes careless mistakes in schoolwork, work, or other activities, often has difficulty sustaining attention in tasks or play activities, (ii) often does not seem to listen when spoken to directly, (iii) often does not follow through on instructions and fails to finish schoolwork, chores, or duties in the workplace (not due to oppositional behavior or failure of comprehension), (iv) often has difficulty organizing tasks and activities, often avoids, dislikes, or is reluctant to engage in tasks that require sustained mental effort (such as schoolwork or homework), (v) often loses things necessary for tasks or activities at school or at home (e.g. toys, pencils, books, assignments), and (vi) often easily distracted by extraneous stimuli, if often forgetful in daily activities. The internal consistency of this scale ranges from 0.63 to 0.72 based on Cronbach’s alpha and its test-retest reliability ranges from 0.58 to 0.77 based on Pearson correlation coefficient.

### Procedure

In the current study, Iranian students were the target population. Participants were practicing students (*n* = 339) selected through subsequent sampling in two stages (outlined above). The study was conducted using a paper-and-pencil method. After agreeing to participate, all participants completed the Computer and Video Game Addiction Scale, Self-control Scale, and Diagnostic Checklist and Self-report Scale. Finally, participants completed the demographics items and students' Grade Point Average (GPA) for two terms as the measure of academic achievement. Verbal instructions indicated that there were no correct answers on any of the scales and that all responses were confidential.

### Ethics

The study procedures were carried out in accordance with the Declaration of Helsinki. The Institutional Review Board of the Islamic Azad University (Department of Educational Psychology) approved the study. All subjects were informed about the study and all provided informed consent. Parental consent was also sought for those younger than 18 years of age.

## RESULTS

The first hypothesis was that there would a relationship between video game addiction, self-control, and academic achievement. This was examined using a regression analysis. Overall, there was a significant relationship between video game addiction, self-control, and academic achievement. As shown in Table 1, “self-control” as the predictor variable was the first variable entered into the model. The correlation between self-control and academic achievement was 0.30 (i.e., self-control only predicted 9.1% of variance related to the students' academic achievement; *R*^2^ = 0.09). In the next step, video game addiction was entered into the model, and the *R*^2^ increased to 0.154 (i.e., 15.4% of variation in the students' academic achievement was explained through a linear relationship with self-control and video game addiction). The contribution of video game addiction was 6.3%. Therefore, each single unit increase in self-control causes an increase of 0.278 units in student academic achievement, and that a single unit increase in video game addiction causes a decrease of 0.252 units in the student academic achievement. As expected, self-control therefore has a positive effect on academic achievement whereas video game addiction has a negative effect.

The relationship between gender differences and video game addiction, self-control, and academic achievement was examined through the multiple regression analysis (hierarchical method). This is summarized in Table 2. Again, there was a significant relationship between gender and academic achievement. When gender was added to the model 3, *R*^2^ increased to 0.263 (i.e., 26.3% of variance related to the student academic achievement was predicted by self-control, video game addiction and gender). Meanwhile the gender contribution rate was almost 10.9% and was statistically significant. Furthermore, the Beta value of this variable was big enough (0.372) to be considered statistically significant. Thus, it can be concluded that the relationship between video game addiction, self-control, and academic achievement differs between male and female students.

The relationship between video game addiction, self-control, academic achievement, and type of student (i.e., normal vs. ADHD) was also examined through multiple regression analysis (hierarchical method). There was a significant effect for type of student (see Table 3). Again, there was a significant relationship between type of student and academic achievement. When type of student was added to the model 3, *R*^2^ increased to 0.156 (i.e., 15.6% of variance related to the student academic achievement was predicted by self-control, video game addiction and type of student). Meanwhile the type of student contribution rate is almost 0.2% which was not statistically significant.

**Table 1:** Coefficients of each variable in the measurement model

Model		*B*	*SE*	β	*t*	p	Δ*R*^2^	*p*
1	Self-control	0.051	0.009	0.278	5.4	<0.001		
2	Video game addiction	–0.055	0.010	–0.252	–4.9	<0.001	0.06	0.01

**Table 2.** Results of hierarchical regression analysis for investigating the relationship between the variables in male and female students

Model		*B*	*SE*	β	*t*	*p*	Δ*R*^2^	*p*
1	Self-control	0.06	0.010	0.301	5.7	<0.001	0.091	0.001
2	Self-control	0.051	0.009	0.278	5.4	<0.001	0.154	0.001
	Video game addiction	–0.06	–0.012	–0.252	–4.9			
3	Self-control	0.08	0.010	0.42	8.04	<0.001	0.263	0.001
	Video game addiction	–0.032	–0.011	–0.144	–2.9			
	Gender	–1.85	–0.268	–0.372	–6.9			

**Table 3.** Coefficients of hierarchical regression analysis for investigating the relationship between the variables in normal and ADHD students

Model		*B*	*SE*	β	*t*	*p*	Δ*R*^2^	*p*
1	Self-control	0.06	0.010	0.301	5.7	<0.001	0.091	0.001
2	Self-control	0.051	0.009	0.278	5.4	<0.001	0.154	0.001
	Video game addiction	–0.06	0.012	–0.252	–4.9				
3	Self-control	0.052	0.006	0.283	8.04	<0.001	0.156	0.001
	Video game addiction	–0.057	0.019	–0.259	–2.9			
	ADHD	–0.052	0.007	–0.353	–6.9			

## DISCUSSION

The findings of this study showed that there was a significantly negative relationship between video game addiction and students' academic achievement, while the relationship between self-control and the academic achievement of these students was significantly positive (i.e., the greater the addiction to video games, the lower the academic achievement). The results are therefore similar to those of Anderson and Dill (2000), Durkin and Barber (2002), and Huge and Gentile (2003). The results cannot demonstrate a causal relationship between video game use, video game addiction, and academic achievement but the results suggest that high involvement in playing video games leaves less time for engaging in academic work.

The findings of this study showed that gender had a significant effect on video game addiction, self-control, and academic achievement (i.e., males were more likely than females to be addicted to video games). This confirms much of the previous research in the area showing that boys spend more of their leisure time playing video games when compared to girls (Griffiths & Hunt, 1995; Buchman & Funk, 1996; Brown et al., 1997; Lucas & Sherry, 2004; Lee, Park & Song, 2005).

There are many likely explanations as to why boys play video games more than girls. First, most video games are designed by males for other males, and even when the games feature strong female characters, they may be highly sexualized and alienate more females than attract them. Second, the socializing procedure is different between males and females. Women are more successful in preventing the emergence of their aggressive behaviors in presence of others, so they may feel more nervous while playing fighting games and are more attracted to more gentle and fantasy games. Furthermore, researchers have used Eagly’s (1987) social role theory in order to explain the reason why boys spend more time playing video games and why they are interested in violent games. This theory is based on the assumption that boys and girls behave according to some predetermined gender clichés and since the content of most of video games are based on competition and violence, they are mostly compatible with the male gender clichés.

According to the findings of this study, the relationship between self-control, video game addiction and academic achievement is significantly different between normal and ADHD students. The present research results supported the findings of Frölich et al. (2009), and Bioulac, Arfi and Bouvard (2008). The commonality between self-control, ADHD and video game addiction is impulsivity. Being unable to mentally concentrate on doing some activities, an impulsive student fails to perform many functions. Enjoying more self-control, a normal student can control the time of playing and avoid playing the video games excessively.

*Limitations and Future research:* There are several limitations to the current study that should be noted. First, the sample size is fairly modest with 326 students. This sample size was smaller than would be desired. Therefore, generalization of its usefulness is limited. Second, because all students included in the analysis were from Iran only, there is no evidence that the findings can be generalized to the population of students in other country. Third, the study employed high school students as participants, and thus, the results of this study may not be generalizable to college students or students older than the age of 18 years (and may additionally be prone to selection and measurement bias). Fourth the cross-sectional design used in this study means that conclusions about cause and effect or sequence of events cannot be made. Finally, the results from this study also raise more general measurement concerns that should be addressed. The questionnaires that used in current study are self-report measures. Previous research suggests that for psychological constructs, self-report measures may not necessarily reflect what a person actually does. It is likely that scores from self-reports of behaviors would be reasonably valid; however, self-reports of behavior may show less consistency with other techniques.

All psychology research is influenced by the participants’ characteristics and developmental stages. Future research should study a more diverse sample of ages, education level, gender, religion, and people from other cultures. Future Studies should use adequate and larger group of students. Research should encompass multiple techniques obtaining data from the same participant (e.g., face-to-face interviews, neurobiological testing, etc.).
